# ﻿Three new species of *Ophiocordyceps* (Hypocreales, Ophiocordycipitaceae) and a new host record for *Hirsutellavermicola* from China

**DOI:** 10.3897/mycokeys.117.144875

**Published:** 2025-05-13

**Authors:** Yu Yang, Yuan-Pin Xiao, Ruvishika S. Jayawardena, Kevin D. Hyde, Somrudee Nilthong, Ausana Mapook, Yong-Zhong Lu, Shu-Qiong Xie, Fatimah Al-Otibi, Xiao Wang, Kang Luo, Li-Ping Luo

**Affiliations:** 1 School of Food and Pharmaceutical Engineering, Guizhou Institute of Technology, Guiyang 550003, China Center of Excellence in Fungal Research, Mae Fah Luang University Chiang Rai Thailand; 2 Center of Excellence in Fungal Research, Mae Fah Luang University, Chiang Rai 57100, Thailand School of Science, Mae Fah Luang University Chiang Rai Thailand; 3 School of Science, Mae Fah Luang University, Chiang Rai, 57100, Thailand School of Food and Pharmaceutical Engineering, Guizhou Institute of Technology Guiyang China; 4 Department of Botany and Microbiology, College of Science, King Saud University, P.O. Box 22452, Riyadh 11495, Saudi Arabia King Saud University Riyadh Saudi Arabia; 5 Guizhou Xishui National Nature Reserve Management Bureau, Xishui 564600, Guizhou, China Guizhou Xishui National Nature Reserve Management Bureau Xishui China

**Keywords:** Morphology, novel taxa, Ophiocordycipitaceae, phylogeny, taxonomy

## Abstract

*Ophiocordyceps*, a genus of invertebrate-pathogenic fungi in the family Ophiocordycipitaceae (order Hypocreales), is globally distributed. Over 300 species have been described and accepted as members of the genus. This study introduces three new *Ophiocordyceps* species from China: *O.liaoningensis*, *O.muscidarum*, and *O.neocommunis*. Remarkably, *O.muscidarum*, hosted on flies (Muscidae, Diptera), is characterized by its larger perithecia and longer secondary ascospores. *Ophiocordycepsneocommunis* is also introduced based on morphological distinctions from closely related species. *Ophiocordycepsliaoningensis* produces dark brown superficial perithecia with an asexual morph at the apex and does not break into part-spores. Phylogenetic analyses using six loci (LSU, ITS, SSU, *tef-1α*, *rpb1*, and *rpb2*) robustly support the placement of these new species within the *Ophiocordyceps*. Additionally, we report a new host record for *Hirsutellavermicola*. Detailed descriptions, illustrations, color photo plates, and a phylogenetic tree are provided.

## ﻿Introduction

Fungi are essential to ecosystems as decomposers, mutualists, and pathogens, contributing to nutrient cycling and decomposition (Hyde et al. 2020, 2024; Niego et al. 2023). Invertebrate-associated fungi, ranging from mutualistic to parasitic, play crucial roles in ecological dynamics across diverse environments (Niego et al. 2023). *Cordycepssensu lato* refers to a group of insect-infecting fungi that are globally distributed, particularly in forest ecosystems (Evans 1982; Sanjuan et al. 2015). This group encompasses four families: Clavicipitaceae, Cordycipitaceae, Ophiocordycipitaceae, and Polycephalomycetaceae (Sung et al. 2007a; Xiao et al. 2023).

*Ophiocordyceps*, initially considered a subgenus of *Cordyceps*, was reclassified as a distinct genus within Ophiocordycipitaceae based on DNA-based phylogenetic analyses (Sung et al. 2007b). With over 300 accepted species, *Ophiocordyceps* is the largest genus within this family and is especially prevalent in tropical and subtropical regions, with Asia being a hotspot of species diversity (Petch 1931, 1933; Sung et al. 2007a; Sanjuan et al. 2015; Spatafora et al. 2015; Hyde et al. 2016, 2017, 2018; Araújo et al. 2018; Luangsa-ard et al. 2018; Khonsanit et al. 2019; Yang et al. 2024). Most species in this genus produce fibrous, tough stromata with cylindrical asci and multi-septate ascospores, exhibiting a wide range of morphologies (Sung et al. 2007a; Quandt et al. 2014; Ban et al. 2015; Xiao et al. 2019). The asexual morphs of *Ophiocordyceps* are primarily characterized by *Hirsutella*, *Hymenostilbe*, *Paraisaria*, and *Syngliocladium* (Sung et al. 2007a; Maharachchikumbura et al. 2015; Mongkolsamrit et al. 2019; Tehan et al. 2023). Historically, *Ophiocordyceps* and *Hirsutella* were considered closely related, with *Hirsutella* initially regarded as a synonym of *Ophiocordyceps* (Evans and Samson 1982; Quandt et al. 2014). However, it was later recognized as valid due to its morphological diversity within Hypocreales (Quandt et al. 2014; Spatafora et al. 2015), and several *Hirsutella* sexual morphs were linked to *Ophiocordyceps*, reinforcing their teleomorphic-anamorphic connection (Simmons et al. 2015).

Species in the genus *Ophiocordyceps* parasitize a wide variety of insect hosts across different orders, demonstrating broad host range and adaptability (Araújo and Hughes 2016; Yang et al. 2021). They infect a diverse range of insect orders, including Blattaria, Coleoptera, Dermaptera, Diptera, Hemiptera, Hymenoptera, Isoptera, Lepidoptera, Megaloptera, Mantodea, Odonata, and Orthoptera, as well as other organisms like protozoans, rotifers, nematodes, and fungi (Ban et al. 2015; Xiao et al. 2019). *Ophiocordyceps* species are known to parasitize insects at various life stages, including larvae, pupae, nymphs, and adults (Kobayasi 1941; Sung et al. 2007a).

During surveys of the taxonomy and diversity of entomopathogenic fungi in various regions of Guizhou and Liaoning provinces, China, eight fungal specimens were collected and preliminarily identified as belonging to *Ophiocordyceps* based on morphological characteristics. Phylogenetic analyses using a multi-locus dataset (LSU, ITS, SSU, *tef-1α*, *rpb1*, and *rpb2*) confirmed their placement within *Ophiocordyceps*. Combined molecular and morphological analyses revealed three previously undescribed species and a new host record of *Hirsutella*, expanding the known fungal diversity in China.

## ﻿Materials and methods

### ﻿Sample collection, macro- and micro-morphological examination

Eight samples were carefully collected from natural habitats in Guizhou and Liaoning provinces, China, from decaying leaf litter or soil, ensuring minimal disturbance to the environment. During collection, relevant metadata, including location, longitude, and latitude, were recorded for each sample (Rathnayaka et al. 2024). The samples were then transported to the laboratory in plastic containers for further examination. In the laboratory, fruiting bodies were sectioned by hand and examined using a stereomicroscope (SMZ 745 and SMZ 800N, Nikon, Tokyo, Japan) to observe macroscopic features. Micromorphological characteristics, including perithecia, asci, ascospores, secondary ascospores, synnemata, conidiophores, phialides, and conidia, were documented using a Nikon DS-Ri2 digital camera connected to a Nikon ECLIPSE microscope.

### ﻿Isolation and material deposition

Pure cultures were obtained by germinating mature ascospores (sexual morphs) or conidia (asexual morphs) or by isolating fungal tissues from surface-sterilized substrates or infected insect hosts, followed by purification (Senanayake et al. 2020). Cultures were grown on potato dextrose agar (PDA, Oxoid, UK) at 25 °C. Living strains were deposited in the Guizhou Culture Collection, China (GZCC), and dried specimens were preserved in the Herbarium of Cryptogams at the Kunming Institute of Botany, Academia Sinica (HKAS). Morphological data were analyzed using Tarosoft (R) v.0.9.7 Image Framework. Photographic plates were prepared and edited using Adobe Photoshop CC 2022 (Adobe Systems, USA). The Faces of Fungi and Index Fungorum numbers were assigned to the new species in accordance with the guidelines of Jayasiri et al. (2015) and https://www.indexfungorum.org/.

### ﻿DNA extraction, PCR amplification, and sequencing

Genomic DNA was extracted from fresh mycelia grown on PDA or directly from dried fungal specimens using the Fungal DNA MiniKit (Biotech, USA), following the directions of the manufacturer. For amplification, six nuclear loci —LSU, ITS, SSU, *tef-1α*, *rpb1*, and *rpb2*—were amplified using their respective primers: LR0R/LR5, ITS5/ITS4, NS1/NS4, EF1-983F/EF1-2218R, CRPB1A/RPB1Cr, and fRPB2-5F/fRPB2-7Cr (Vilgalys and Hester 1990; White et al. 1990; Hopple 1994; Castlebury et al. 2004; Sung et al. 2007b). PCR amplifications were conducted with an initial denaturation at 98 °C for 2 minutes, followed by 40 cycles of 98 °C for 10 seconds, 55 °C for 1 minute, and 72 °C for 30 seconds, with a final extension step at 72 °C for 2 minutes. The PCR products were verified by electrophoresis on a 1% agarose gel stained with ethidium bromide in a TBE buffer. The PCR products were purified and sequenced using the Sanger method at Shenggong Biological Engineering Co. (Shanghai, China). All newly generated sequences were uploaded to GenBank, and the corresponding accession numbers are provided in Table 1.

**Table 1.** Names, voucher numbers, references, and corresponding GenBank accession numbers of the taxa used in the phylogenetic analysis of this study.

Taxa names	Specimen/ Strain number	GenBank accession numbers						References
		LSU	ITS	SSU	*tef-1α*	*rpb1*	*rpb2*	
*Hirsutellagigantea*	ARSEF 30	JX566977			JX566980	KM652034	—	Simmons et al. 2015
*Hirsutellaguyana*	ARSEF 878	KM652111	KM652158	KM652068	KM651994	KM652035	—	Simmons et al. 2015
*Hirsutellalecaniicola*	ARSEF 8888	KM652114	KM652162	KM652071	KM651998	KM652038	—	Simmons et al. 2015
*Hirsutellaminnesotensis*	SB3612	—	EF194145	—	—	—	—	Balazy et al. 2008
*Hirsutellaminnesotensis*	CBS.115627	—	DQ078757	—	—	—	—	Xiang et al. 2010
*Hirsutellanodulosa*	ARSEF 5473	KM652117	KM652165	KM652074	KM652000	KM652040	—	Simmons et al. 2015
*Hirsutellaradiata*	ARSEF 1369	KM652119	—	KM652076	KM652002	KM652042	—	Simmons et al. 2015
*Hirsutellarhossiliensis*	ARSEF 2931	KM652121	KM652168	KM652078	KM652004	KM652043	—	Simmons et al. 2015
*Hirsutellastrigosa*	ARSEF 2197	KM652129	KM652175	KM652085	KM652012	KM652050	—	Simmons et al. 2015
*Hirsutellathompsonii*	ARSEF 257	KM652136	KM652182	—	KM652019	KM652054	—	Simmons et al. 2015
*Hirsutellathompsonii*	ARSEF 414	KM652139	KM652184	—	KM652021	KM652056	—	Simmons et al. 2015
*Hirsutellavermicola*	AS3.7879	—	DQ345581	—	—	—	—	Xiang et al. 2006
*Hirsutellavermicola*	CGMCC 3.7877^T^	—	NR_137547	—	—	—	—	Xiang et al. 2006
*Hirsutellavermicola*	AS3.7878	—	DQ345592	—	—	—	—	Xiang et al. 2006
***Hirsutellavermicola***	**HKAS 132167**	**PQ423697**	**PQ423678**	**PQ424974**	**PQ569874**	**PQ569888**	**PQ569904**	**This study**
***Hirsutellavermicola***	**HKAS 132168**	**PQ423698**	**PQ423679**	**PQ424975**	**PQ569875**	**PQ569889**	**PQ569905**	**This study**
*Hirsutellaversicolor*	ARSEF 1037	KM652150	—	KM652102	KM652029	KM652063	—	Simmons et al. 2015
*Hymenostilbedipterigena*	NHJ12170	—	GU723771	—	GU797127	—	—	Luangsa-ard et al. 2011
*Ophiocordycepslongistipes*	HKAS126185^T^	OR015966	OR015960	OR082947	OR030531	OR062225	—	Fan et al. 2024
*Ophiocordycepsacicularis*	OSC 110988	EF468804	—	EF468951	EF468745	EF468853	—	Sung et al. 2007b
*Ophiocordycepsacicularis*	OSC 110987	EF468805	—	EF468950	EF468744	EF468852	—	Sung et al. 2007b
*Ophiocordycepsagriotidis*	ARSEF 5692	DQ518754	JN049819	DQ522540	DQ522322	DQ522368	DQ522418	Spatafora et al. 2007
*Ophiocordycepsannulata*	CEM 303	—	—	KJ878915	KJ878962	KJ878995	—	Quandt et al. 2014
*Ophiocordycepsaphodii*	ARSEF 5498	DQ518755	—	DQ522541	DQ522323	—	DQ522419	Spatafora et al. 2007
*Ophiocordycepsappendiculata*	NBRC 106959	JN941412	JN943325	JN941729	AB968578	JN992463	AB968540	Ban et al. 2015
*Ophiocordycepsasiatica*	BCC 30516^T^	MH753675	MH754722	—	MK284263	MK214105	MK214091	Tasanathai et al. 2019
*Ophiocordycepsbidoupensis*	YHH 20036^T^	—	—	OK571396	OK556893	OK556897	OK556899	Zou et al. 2022
*Ophiocordycepsbispora*	KVL 606	AF009654	—	KX713641	—	KX713716	—	Araújo et al. 2018
*Ophiocordycepsborealis*	MFLU 18-0163^T^	MK863052	MK863252	MK863045	MK860190	—	—	Zha et al. 2021
*Ophiocordycepsbrunneiperitheciata*	BCC 49312	MF614660	—	—	MF614642	—	MF614686	Luangsa-ard et al. 2018
*Ophiocordycepsbrunneipunctata*	OSC 128576	DQ518756	——	DQ522542	DQ522324	DQ522369	DQ522420	Spatafora et al. 2007
*Ophiocordycepsbrunneirubra*	BCC 14478^T^	MH753688	MH754734	—	GU797122	MK751466	MK214102	Tasanathai et al. 2019
*Ophiocordycepsbrunneirubra*	BCC 14384	MH753690	MH754736	—	GU797121	MK751465	MK751468	Tasanathai et al. 2019
*Ophiocordycepsbrunneirubra*	BCC 14477	MH753689	MH754735	—	GU797123	MK751467	MK214103	Tasanathai et al. 2019
*Ophiocordycepscamponoti-hippocrepidis*	HIPPOC^T^	KX713597	—	KX713655	KX713673	KX713707	—	Araújo et al. 2018
*Ophiocordycepscamponoti-nidulantis*	NIDUL2^T^	KX713611	—	KX713640	KX713669	KX713717	—	Araújo et al. 2018
*Ophiocordycepscamponoti-rufipedis*	G108	KX713594	—	KX713659	KX713679	KX713704	—	Araújo et al. 2018
*Ophiocordycepsclavata*	NBRC 106961	JN941414	JN943327	JN941727	AB968586	JN992461	AB968547	Ban et al. 2015
*Ophiocordycepscommunis*	BCC 1842	MH753680	MH754726	—	MK284266	MK214110	MK214096	Tasanathai et al. 2019
*Ophiocordycepscommunis*	NHJ 1131	MH753679	MH754725	—	MK284267	MK214109	MK214095	Tasanathai et al. 2019
*Ophiocordycepscommunis*	NHJ 10673^T^	MH753681	MH754727	—	MK284268	MK214111	MK214097	Tasanathai et al. 2019
*Ophiocordycepscurculionum*	OSC 151910	KJ878885	—	KJ878918	—	KJ878999	—	Quandt et al. 2014
*Ophiocordycepsdipterigena*	HUA 186102	KJ917568	—	KC610787	—	KF658664	KC610715	Quandt et al. 2014
*Ophiocordycepsdipterigena*	OSC 151911	KJ878886	—	KJ878919	KJ878966	KJ879000	—	Quandt et al. 2014
*Ophiocordycepsdipterigena*	OSC 151912	KJ878887	—	KJ878920	KJ878967	KJ879001	—	Quandt et al. 2014
*Ophiocordycepsdipterigena*	MRCIF71	—	EU573346	—	—	—	—	Freire 2015
*Ophiocordycepsdipterigena*	MY621	—	GU723764	—	GU797126	—	—	Luangsa-ard et al. 2011
*Ophiocordycepsferruginosa*	NBRC 101743	—	AB968405	—	—	—	—	Sung et al. 2007b
*Ophiocordycepsformosana*	TNM F13893	—	—	KJ878908	KJ878956	KJ878988	KJ878943	Quandt et al. 2014
*Ophiocordycepsforquignonii*	OSC 151908	KJ878889	KJ878922	—	—	KJ879003	KJ878947	Quandt et al. 2014
*Ophiocordycepsforquignonii*	OSC 151902	KJ878876	KJ878912	—	—	KJ878991	KJ878945	Quandt et al. 2014
*Ophiocordycepsfurcatosubulata*	YFCC 904^T^	MT774222	—	MT774215	MT774243	MT774229	MT774236	Wang et al. 2021
*Ophiocordycepsfusiformis*	BCC 93025^T^	MZ675422	MZ676743	—	MZ707849	MZ707855	MZ707805	Tasanathai et al. 2022
*Ophiocordycepsglobiceps*	MFLUCC 18-0495	MH725829	MH725815	MH725811	MH727387	—	—	Xiao et al. 2019
*Ophiocordycepsglobiceps*	MFLU 18-0661^T^	MH725830	MH725816	MH725812	MH727388	—	—	Xiao et al. 2019
*Ophiocordycepsglobiperitheciata*	HKAS126130^T^	OR015968	OR015963	OR082950	OR030532	OR119834	—	Fan et al. 2024
*Ophiocordycepsglobosa*	BCC 93023^T^	MZ675419	MZ676740	—	MZ707846	MZ707861	—	Tasanathai et al. 2022
*Ophiocordycepsgracillima*	HUA 186132	KC610768	KF937353	—	KC610744	KF658666	—	Sanjuan et al. 2015
*Ophiocordycepshemisphaerica*	FLOR 59525^T^	—	KX197233	—	—	—	—	Hyde et al. 2016
*Ophiocordycepshemisphaerica*	FLOR 59542	—	KX197234	—	—	—	—	Hyde et al. 2016
*Ophiocordycepshemisphaerica*	FLOR 59553	—	KX197235	—	—	—	—	Hyde et al. 2016
*Ophiocordycepsisopterorum*	MY 12376.01	MZ675420	MZ676741	—	MZ707847	MZ707859	MZ707803	Tasanathai et al. 2022
*Ophiocordycepsisopterorum*	BCC 93042^T^	MZ675421	MZ676742	—	MZ707848	—	—	Tasanathai et al. 2022
*Ophiocordycepskhokpasiensis*	BCC 1764	MH753684	MH754730	—	MK284271	MK214114	MK214098	Tasanathai et al. 2019
*Ophiocordycepskhokpasiensis*	BCC 48072	MH753682	MH754728	—	MK284269	MK214112	—	Tasanathai et al. 2019
*Ophiocordycepskhokpasiensis*	BCC 48071^T^	MH753683	MH754729	—	MK284270	MK214113	—	Tasanathai et al. 2019
*Ophiocordycepskimflemingiae*	SJS4Oph	MH536516	—	MH536517	MN785130	MN785132	—	Araújo et al. 2018
*Ophiocordycepskonnoana*	EFCC 7315	—	—	EF468959	EF468753	EF468861	EF468916	Sung et al. 2007b
*Ophiocordycepskuchinaraiensis*	BCC 95830^T^	OQ627397	OQ627396	—	OQ625474	—	OQ625475	Tasanathai et al. 2019
*Ophiocordycepslacrimoidis*	FLOR 59552^T^	—	KX197231	—	—	—	—	Freire 2015
***Ophiocordycepsliaoningensis***	**HKAS 132185**	**PQ423690**	**PQ423671**	—	**PQ569869**	**PQ569883**	**PQ569897**	**This study**
***Ophiocordycepsliaoningensis***	**HKAS 132189**	**PQ423691**	**PQ423672**	**PQ424968**	**PQ569870**	**PQ569884**	**PQ569898**	**This study**
***Ophiocordycepsliaoningensis***	**HKAS 132276** ^T^	**PQ423692**	**PQ423673**	**PQ424969**	**PQ569871**	**PQ569885**	**PQ569899**	**This study**
*Ophiocordycepsmacroacicularis*	NBRC 100685^T^	AB968416	AB968400	AB968388	AB968574	—	AB968536	Ban et al. 2015
*Ophiocordycepsmosingtoensis*	BCC 30904	MH753686	MH754732	—	MK284273	MK214115	MK214100	Tasanathai et al. 2019
*Ophiocordycepsmosingtoensis*	BCC 36921^T^	MH753685	MH754731	—	MK284272	MK214116	MK214099	Tasanathai et al. 2019
***Ophiocordycepsmuscidarum***	**HKAS 132178** ^T^	**PQ423695**	**PQ423676**	**PQ424972**	**PQ675604**	—	**PQ569900**	**This study**
***Ophiocordycepsmuscidarum***	**HKAS 132275**	**PQ423696**	**PQ423677**	**PQ424973**	**PQ675605**	—	**PQ569901**	**This study**
***Ophiocordycepsneocommunis***	**HKAS 132236** ^T^	**PQ423693**	**PQ423674**	**PQ424970**	**PQ569872**	**PQ569886**	**PQ569902**	**This study**
***Ophiocordycepsneocommunis***	**GZCC 24-0158**	**PQ423694**	**PQ423675**	**PQ424971**	**PQ569873**	**PQ569887**	**PQ569903**	**This study**
*Ophiocordycepsneovolkiana*	OSC 151903	KJ878896	—	KJ878930	KJ878976	KJ879010	—	Quandt et al. 2014
*Ophiocordycepsovatospora*	YHH 2206001^T^	OP295113	OP295105	OP295110	OP313801	OP313803	OP313805	Tang et al. 2022
*Ophiocordycepspseudocommunis*	BCC 16757^T^	MH753687	MH754733	—	MK284274	MK214117	MK214101	Tasanathai et al. 2019
*Ophiocordycepspseudocommunis*	NHJ 12581	EF468831	—	—	EF468775	—	EF468930	Sung et al. 2007b
*Ophiocordycepspseudocommunis*	NHJ 12582	EF468830	—	—	EF468771	—	EF468926	Sung et al. 2007b
*Ophiocordycepspseudorhizoidea*	BCC 86431^T^	MH753674	MH754721	—	MK284262	MK751469	MK214090	Tasanathai et al. 2019
*Ophiocordycepspseudorhizoidea*	BCC 48879	MH753673	MH754720	—	MK284261	MK214104	MK214089	Tasanathai et al. 2019
*Ophiocordycepsradiciformis*	BCC 93036^T^	MZ675425	MZ676746	—	MZ707852	MZ707857	MZ707808	Tasanathai et al. 2022
*Ophiocordycepsrobertsii*	YHORZT007	KC561978	—	KC561978	KC561979	KC561980	—	Sung et al. 2007b
*Ophiocordycepsrubiginosiperitheciata*	NBRC 100946	JN941436	JN943341	JN941705	AB968581	JN992439	AB968543	Ban et al. 2015
*Ophiocordycepsryogamiensis*	NBRC 101751	KF049633	—	KF049614	KF049688	KF049650	—	Sanjuan et al. 2015
*Ophiocordycepssatoi*	J19	KX713601	—	KX713650	KX713684	KX713710	—	Araújo et al. 2018
*Ophiocordycepssinensis*	EFCC7287	EF468827	JN049854	EF468971	EF468767	EF468874	EF468924	Sung et al. 2007b
*Ophicordyceps* sp.	FMF147	—	KX197238	—	—	—	—	Freire 2015
*Ophiocordycepsspataforae*	OSC 128575	EF469079	JN049845	EF469126	EF469064	EF469093	EF469110	Sung et al. 2007b
*Ophiocordycepsspicatus*	MFLU 18-0164^T^	MK863054	MK863254	MK863047	MK860192	—	—	Tasanathai et al. 2019
*Ophiocordycepsstylophora*	OSC 111000	DQ518766	JN049828	DQ522552	DQ522337	DQ522382	DQ522433	Spatafora et al. 2007
*Ophiocordycepstermiticola*	BCC 1920^T^	MH753678	MH754724	—	MK284265	MK214108	MK214094	Tasanathai et al. 2019
*Ophiocordycepstermiticola*	BCC 1770	MH753677	GU723780	—	MK284264	MK214107	MK214093	Tasanathai et al. 2019
*Ophiocordycepsunilateralis*	SERI1	KX713626	—	KX713628	KX713675	KX713730	—	Araújo et al. 2018
*Ophiocordycepsunituberculata*	YHH HU1301^T^	—	KY923211	KY923213	KY923215	KY923217	—	Wang et al. 2018
*Ophiocordycepsunituberculata*	YFCC HU1301	—	KY923212	KY923214	KY923216	KY923218	KY923220	Wang et al. 2018
*Ophiocordycepsvariabilis*	ARSEF 5365	DQ518769	—	DQ522555	DQ522340	DQ522386	DQ522437	Spatafora et al. 2007
*Ophiocordycepsvariabilis*	OSC 111003	EF468839	—	EF468985	EF468779	EF468885	EF468933	Sung et al. 2007b
*Ophiocordycepsxuefengensis*	GZUH2012HN14^T^	—	KC631800	KC631786	KC631791	KC631796	—	Wen et al. 2013
*Ophiocordycepsmelolonthae*	OSC 110993	DQ518762	—	DQ522548	DQ522331	DQ522376	—	Spatafora et al. 2007
*Tolypocladiuminflatum*	OSC 71235	EF469077	JN049844	EF469124	EF469061	EF469090	EF469108	Sung et al. 2007b
*Tolypocladiumophioglossoides*	NBRC 106332	JN941409	JN943322	JN941732	—	JN992466	—	Schoch et al. 2012

Note: The symbol “—” means the sequence unavailability, and sequences newly generated for this study are in bold. “T” represents the type strain.

### ﻿Phylogenetic analyses

All newly generated sequences were assembled using SeqMan (Clewley 1995). Reference taxa for phylogenetic analyses were identified via BLAST searches (https://blast.ncbi.nlm.nih.gov/Blast.cgi) and referenced from previous studies (Table 1). Individual sequences were aligned with MAFFT v.7 (https://mafft.cbrc.jp/alignment/server/) and refined using TrimAl (Capella-Gutiérrez et al. 2009; Katoh and Standley 2013). Alignments were manually inspected and adjusted in BioEdit (Hall et al. 2011). Phylogenetic relationships were conducted using maximum likelihood (ML) and Bayesian inference (BI). *Tolypocladiuminflatum* (OSC 71235) and *T.ophioglossoides* (NBRC 106332) were selected as outgroup taxa (Xiao et al. 2019; Yang et al. 2021, 2024).

Maximum likelihood analyses were conducted using IQ-TREE 2 under partitioned models and 1,000 bootstrap replicates to assess branch support (Minh et al. 2020). ModelFinder was used to select the optimal substitution model for each locus (Kalyaanamoorthy et al. 2017). Bayesian inference was performed using MrBayes v.3.1.2 (Ronquist et al. 2012), employing Markov Chain Monte Carlo (MCMC) sampling with six parallel chains, run for 1,000,000 to 5,000,000 generations. Convergence was determined by achieving a standard deviation of split frequencies below 0.01, with trees sampled every 1,000 generations. The first 25% of sampled trees (25,000) were discarded as burn-in, and the remaining trees were used to calculate posterior probabilities (PP). The resulting maximum likelihood tree was visualized using FigTree v.1.4.0 (http://tree.bio.ed.ac.uk/software/figtree/).

### ﻿Phylogenetic analysis results

**Figure 1.**
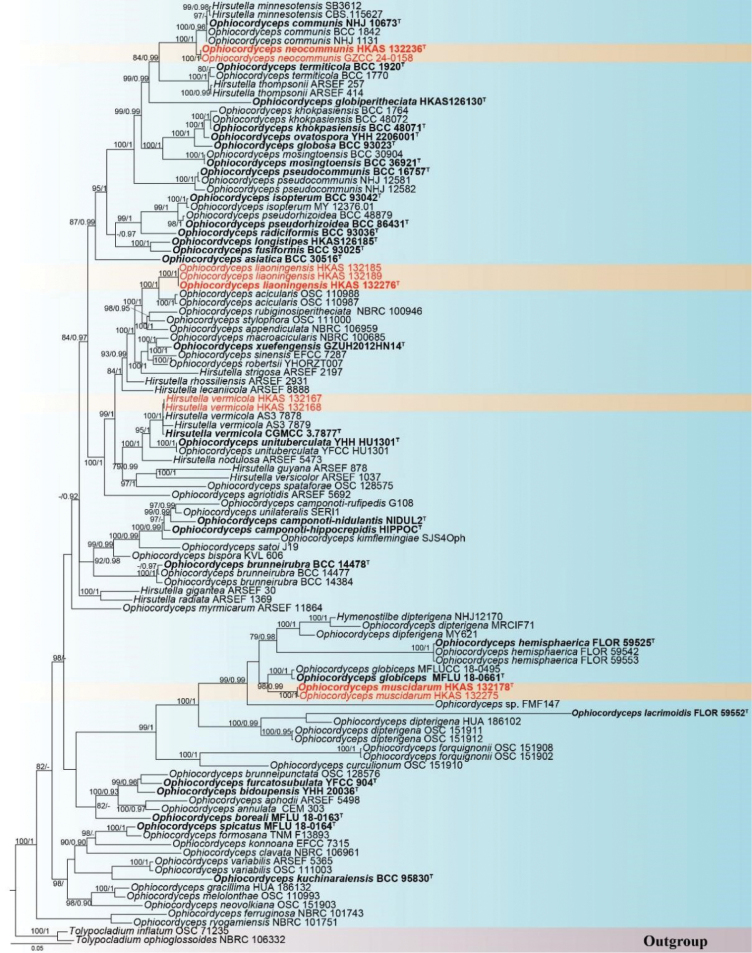
A phylogram constructed using a maximum likelihood (ML) analysis with RAxML based on concatenated LSU, ITS, SSU, *tef-1α*, *rpb1*, and *rpb2* sequence data. *Tolypocladiuminflatum* and *T.ophioglossoides* were employed as outgroup taxa. Nodes with maximum likelihood bootstrap values ≥ 75% and Bayesian posterior probabilities ≥ 0.90 are indicated on the phylogram. Type strains are in bold and marked with ^T^, and the new species are in red.

Reference sequences (Table 1) were downloaded from NCBI GenBank based on previous studies (Sung et al. 2007a; Ban et al. 2015; Sanjuan et al. 2015; Xiao et al. 2019; Tang et al. 2022). In this study, we collected eight specimens and one strain, from which 51 new sequences were generated, including 9 LSU, 9 ITS, 8 SSU, 9 *tef-1α*, 7 *rpb1*, and 9 *rpb2*. Six loci—LSU, ITS, SSU, *tef-1α*, *rpb1*, and *rpb2*—were used to determine the phylogenetic placement of the new collections. The concatenated matrix consisted of 108 taxa with a total of 4,653 base pairs (bp) across the loci (LSU: 1–832 bp; ITS: 833–1,319 bp; SSU: 1,319–2,258 bp; *tef-1α*: 2,259–3,152 bp; *rpb1*: 3,153–3,806 bp; *rpb2*: 3,807–4,653 bp). This matrix was deposited in TreeBASE (accession URL: http://purl.org/phylo/treebase/phylows/study/TB2:S31902). Single-locus analyses were performed to compare the topologies and clade stabilities. The optional models selected by ModelFinder were as follows: LSU: TNe+I, ITS: TIM3e+G4, SSU: K2P, *tef-1α*: TN+F+G4, *rpb1*: TNe+G4, *rpb2*: TNe+I. The ML and BI analyses produced nearly congruent topologies; therefore, the best-scoring ML tree is presented in Fig. 1. *Ophiocordycepsmuscidarum* (HKAS 132178 and HKAS 132275) was sister to *O.globiceps* (MFLU 18-0661 and MFLUCC 18-0495) and formed a strongly supported monophyletic lineage (100% MLBP, 1.00 PP). *Ophiocordycepsneocommunis* was sister to *O.communis* (BCC 1842, NHJ 1131, and NHJ 10676) and *Hirsutellaminnesotensis* (SB3612 and CBS.115627) in the phylogenetic tree, with strong support (100% MLBP, 1.00 PP). *Ophiocordycepsliaoningensis* (HKAS 132276, HKAS132189, HKAS 132185) was sister to *O.acicularis* (OSC 110988 and OSC 110987) and also formed a strongly supported monophyletic lineage (100% MLBS/1.00 BIPP). Two collections (HKAS 132167 and HKAS 132168) were nested with *Hirsutellavermicola* (AS3.7878, AS3.7879, and CGMCC 3.7877) without branch length support. Based on these phylogenetic analyses, the collections were identified as three new species, namely *Ophiocordycepsliaoningensis*, *O.muscidarum*, *and O.neocommunis*, as well as a new host record for *Hirsutellavermicola*.

## ﻿Taxonomy

### ﻿ Ophiocordyceps liaoningensis

Taxon classificationFungiHypocrealesOphiocordycipitaceae

Y. P. Xiao, K.D. Hyde & Y. Yang sp. nov.

3A880B70-C4AC-5A1E-91B5-09090F1FF5C3

Index Fungorum: IF902881

Facesoffungi Number: FoF16768

Fig. 2

#### Etymology.

The epithet “*liaoningensis*” refers to the type location “Liaoning Province, China”.

#### Holotype.

China • Liaoning Province, Tieling City, Xifeng County, at 377 masl, 42.653°N, 124.452°E, parasitic on the larva of Coleoptera, buried in soil, 20 July 2023, Yuanpin Xiao (HKAS 132276).

#### Description

**(HKAS 132276). *Parasitic*** on the larva of Coleoptera (Elateridae), buried in the soil. ***Sexual morph*: *Host*** 2.0 cm long, 2–4 mm wide, without hyphae on the surface. ***Stromata*** 5–6 cm long, 1–3 mm wide, single, stipitate, cylindrical, pale brown, arising from the host head. ***Fertile head*** 1–2 cm long, 2–3 mm wide, with superficial perithecia along the surface of the stipe, cylindrical, dark brown, with asexual morph at the apex. ***Perithecia*** 310–415 × 170–290 μm (*x*– = 362.5 × 230 µm, n = 20), superficial, dark brown, ovoid to flask-shaped, thick-walled. ***Peridium*** 30–50 µm (*x*– = 40 µm, n = 30) wide, two layers, ***textura angularis*** outer layer to ***textura porrecta*** inner layer, outer layer brownish, inner layer hyaline. ***Asci*** 205–255 × 7–11 μm (*x*– = 230 × 9 µm, n = 30), 8-spored, cylindrical, hyaline, with a thin apex. ***Apical cap*** 5.5–6.5 × 3.5–4.7 μm (*x*– = 6 × 4.1 µm, n = 40), hyaline. ***Ascospores*** 150–200 × 2–4 μm (*x*– = 175 × 3 µm, n = 60), multiseptate, slender filiform, not breaking into secondary ascospores. ***Asexual morph*: *Hymenostilbe***-like. ***Synnemata*** 0.5 cm long, 0.5–1 mm wide, single, cylindrical, light brown, tapering upwards. ***Conidiophores*** 10–28 µm wide (*x*– = 19 µm, n = 40), usually simple, branched or unbranched, septate, hyaline, bearing conidia. ***Phialide*** 15–33 × 3.5–6.5 µm (*x*– = 24 × 5 µm, n = 40), polyblastic, hyaline, clavate or bottle-shaped, forming a dense palisade layer covering the synnemata. ***Conidia*** 5–9 × 4.2–6.4 µm (*x*– = 7 × 5.3 µm, n = 60), 1-celled, hyaline, ovoid or subglobose, developing along the tip of the phialide.

#### Additional material.

China • Liaoning Province, Tieling City, Xifeng County, at 394 masl, 42.656°N, 124.449°E, 20 July 2023, Yuanpin Xiao, TL2356 (HKAS 132189, paratype); China • Liaoning Province, Tieling City, Xifeng County, at 368 masl, 42.654°N, 124.454°E, 20 July 2023, Yuanpin Xiao, TL01 (HKAS 132185).

**Figure 2.**
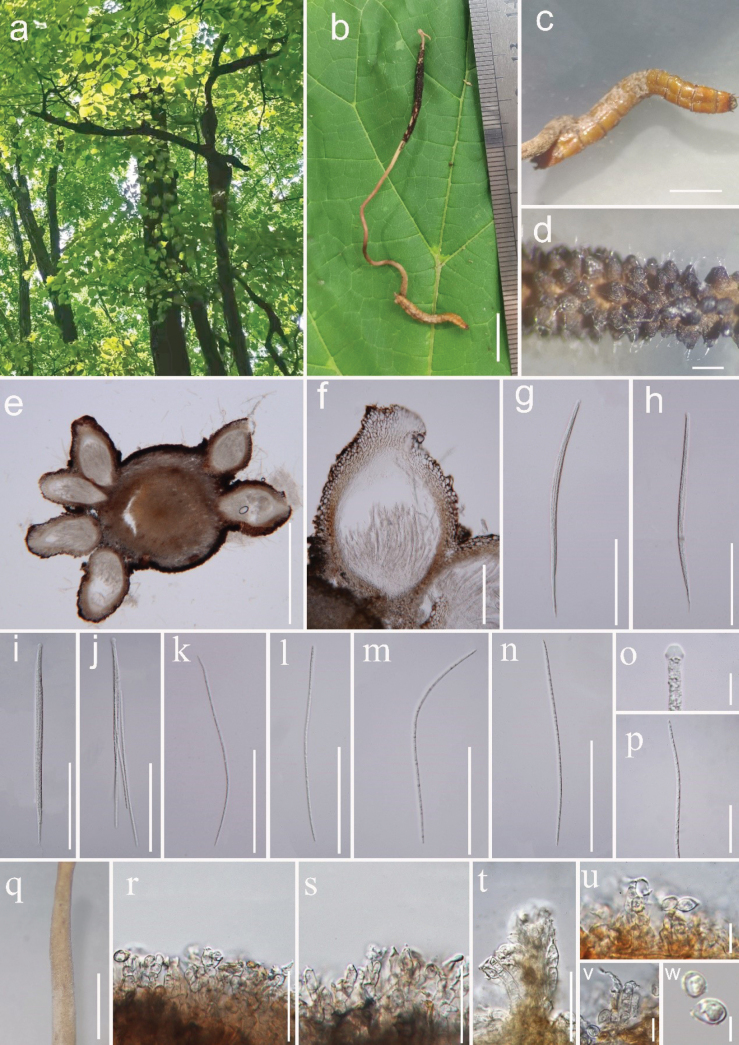
*Ophiocordycepsliaoningensis* (HKAS 132276, holotype) **a** habitat **b** overview of the host and stroma **c** host (larva of Elateridae) **d** fertile part on stroma showing superficial perithecia **e–f** cross section of perithecia **g–i** asci **j–n, p** ascospores **o** apical cap **q** synnema **r–t** conidiophores **u–t** phialide **w** conidia. Scale bars: 1 cm (**b**); 0.5 cm (**c, q**); 0.1 cm (**d**); 500 μm (**e**); 100 μm (**f–n**); 30 μm (**r–t, p**); 10 μm (**u, v**); 5 μm (**w**).

#### Notes.

*Ophiocordycepsliaoningensis* clustered with *O.acicularis* in the phylogenetic tree with support (100% MLBP, 1.00 PP) (Fig. 1). Nucleotide differences of LSU, ITS, *tef-1α*, *rpb1*, and *rpb2* sequences of *O.acicularis* are 2.09% (17/810), 6.74% (34/504), 6.77% (63/931), 5.61% (39/695), and 9.44% (105/1112), respectively. Morphologically, *Ophiocordycepsacicularis* produces shorter perithecia (280 × 250 μm vs. 310–415 μm), longer asci (260–290 × 7–10 μm vs. 205–255 × 7–11 μm), and longer ascospores (150–240 × 3–4 μm vs. 150–200 × 2–4 μm), compared to *O.liaoningensis* (Petch 1933). *Ophiocordycepsagriotidis* is similar to *O.liaoningensis* in having superficial perithecia and multiseptate ascospores. However, *O.agriotidis* differs by producing larger perithecia (380–550 × 280–350 μm vs. 310–415 × 170–290 μm) and longer asci (260–280 × 8.5–9.0 μm vs. 205–255 × 7–11 μm) (Kobayasi and Shimizu 1980). Furthermore, the phylogenetic tree clearly distinguishes *O.agriotidis* from *O.liaoningensis* (Fig. 1). Zha et al. (2021) provided the first comprehensive review of wireworm-infecting *Cordyceps* sensu lato species, documenting 27 species within *Ophiocordyceps*. These fungi are phylogenetically distinct from *O.liaoningensis*. However, eight species were described solely based on morphological characteristics, highlighting the need for further molecular studies to clarify their taxonomic status (Zha et al. 2021). In contrast, *Ophiocordycepsliaoningensis* is characterized by superficial perithecia, multiseptate ascospores that do not break into part-spores, bottle-shaped phialides, and ovoid or subglobose conidia (Table 2). Both morphological observation and phylogenetic analyses of combined LSU, ITS, SSU, *tef-1α*, *rpb1*, and *rpb2* sequence data support that this fungus is a distinctive species in *Ophiocordyceps*.

**Table 2.** Synopsis of a comparison of *O.liaoningensis* sp. nov. and its closely related *Ophiocordyceps* species.

Species	Distribution	Stromata (mm)	Perthecia (μm)	Asci (μm)	Ascospores (μm)	Secondary ascospores (μm)	References
*O.asyuensis*	Japan	7–15 × 0.5	Superficial, 600–650 × 450	400–500 × 7–8	—	7–8 × 1.5	Kobayasi and Shimizu 1982
*O.elateridicola*	Japan	70 × 3	Immersed, 400–450 × 120–150	4–3 in diam	—	3–4 × 1	Kobayasi and Shimizu 1982
*O.falcatoides*	Japan	17–24 × 0.8–1	Superficialia, 350–400 × 200–250	150–175 × 7–8	125–150 × 2.5–3	9–11 × 3	Kobayasi and Shimizu 1980
*O.larvicola*	France	5–6	Ovoid, small, purplish brown.			5–8, formed by three globules	Quélet 1879
***O.liaoningensis***	**China**	**50–60 × 1–3**	**Superficial, 310–415 × 170–290**	**205–255 × 7–11**	**150–200 × 2–4**	—	**This study**
*O.nigripoda*	Japan	25–30 × l	Immersed, 320–350 × 150–180 μ	115–140 × 10	57–90 × 3.3–5 septatae	—	Kobayasi and Shimizu 1982
*O.rubripunctata*	Ghana	60 × 0.5–1.5	Embedded, 360–600 × 145–325	Cylindrical with a 2–3 thick	Filiform	6–9 × 1.0–2.5	Samson et al. 1982
*O.salebrosa*	Panama Canal Zone	35–40	Immersed, 840–1200 × 240–300	600–660 × 64	Filiformibus	6–10 × 1–1.5	Mains 1947
*O.subflavida*	Venezuela	2–4	500–600 × 150–200	225–300 × 3–4	Filiform	—	Mains 1959

The symbol “—” means that the data is unavailable.

### ﻿ Ophiocordyceps muscidarum

Taxon classificationFungiHypocrealesOphiocordycipitaceae

Y. P. Xiao, K.D. Hyde & Y. Yang sp. nov.

4F6D2CD5-57E4-5FAB-96C5-80A22D51A522

Index Fungorum: IF902879

Facesoffungi Number: FoF16766

Fig. 3

#### Etymology.

The epithet “*muscidarum*” refers to its host belonging to the family Muscidae (Diptera).

#### Holotype.

China • Liaoning Province, Tieling City, Xifeng County, at 356 masl, 42.663°N, 124.482°E, parasitic on the fly (Muscidae, Diptera), collected on a tree stem, 20 July 2023, Yuanpin Xiao (HKAS 132178).

#### Description

**(HKAS 132178). *Parasitic*** on flies (Muscidae, Diptera), collected on a tree stem. ***Host*** 6–8 mm long, 3–5 mm wide, without hyphae on the surface. ***Sexual morph*: *Stromata*** 5–7 mm long, 1–4 mm diam., one or several growing from the host prothorax, stipitate, capitate, unbranched, cinnamon to pale yellow. ***Stipe*** 3–5 mm long, 1–2 mm diam, cinnamon to pale brown, cylindrical, with a fertile apex. ***Fertile head*** hemispherical to globose, 1.5–4 mm, cinnamon to pale yellow, single. ***Perithecia*** 570–760 × 190–310 μm (*x*–= 665 × 250 µm, n = 30), immersed, ovoid to flask-shaped, thick-walled. ***Peridium*** 30–50 µm (*x*–= 40 µm, n = 60) wide, hyaline, three layers: outer layer to ***textura porrecta***, middle layer ***textura prismatica***, inner layer ***textura angularis***. ***Asci*** 280–430 × 5.4–7.5 μm (*x*–= 355 × 6.5 µm, n = 60), 8-spored, hyaline, cylindrical, with a thick apex. ***Apical cap*** 5.2–7.6 × 4.4–5.2 μm (*x*–= 6.4 × 4.8 µm, n = 60), thick, hyaline. ***Ascospores*** as long as asci, filiform, hyaline, easily breaking into part-spores. ***Secondary ascospores*** 7–10.5 × 1.6–2.5 μm (*x*–= 8.8 × 2.1 µm, n = 60) fusiform, 1-celled, mostly straight, hyaline, smooth-walled. ***Asexual morph*** Not observed in natural substrates.

#### Additional material.

China • Liaoning Province, Tieling City, Xifeng County, at 374 masl, 42.665°N, 124.487°E, parasitic on the fly (Muscidae, Diptera), collected on a tree stem, 20 July 2023, Yuanpin Xiao TL2378 (HKAS 132275, paratype).

**Figure 3.**
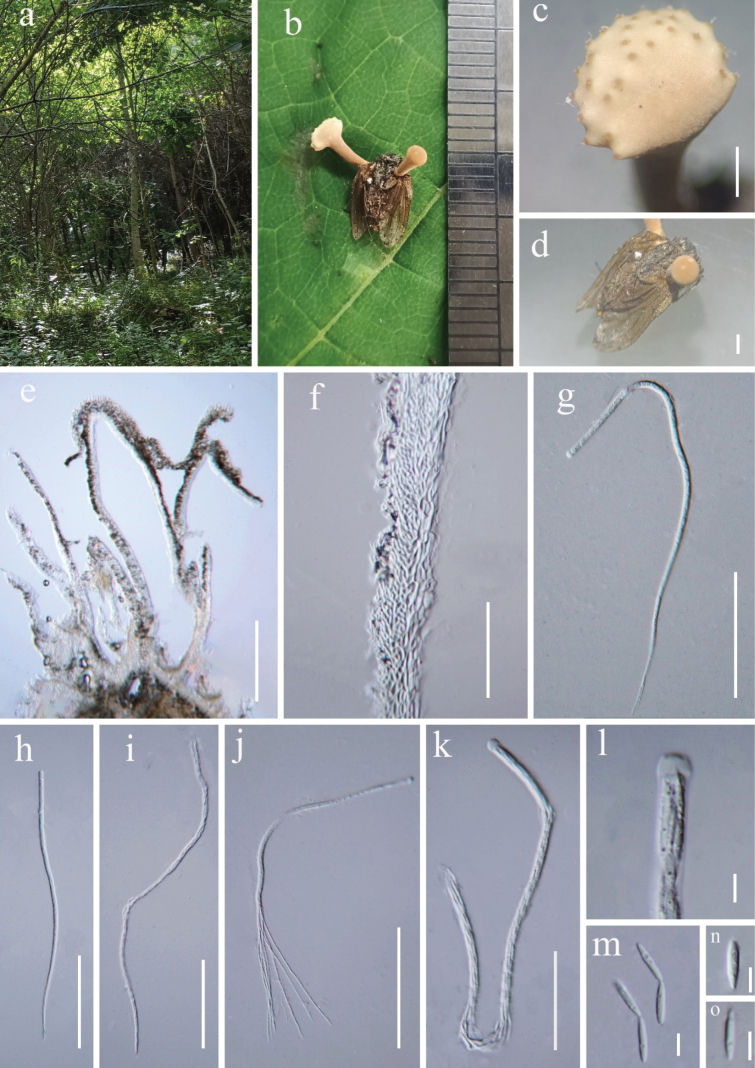
*Ophiocordycepsmuscidarum* (HKAS 132178, holotype) **a** habitat **b, c** stromata **d** host (Muscidae, Diptera) **e** perithecia **f** peridium **g-j** asci **k** ascus apical cap **l–n** secondary ascospores. Scale bars: 0.1 cm (**c, d**); 200 μm (**e**); 50 μm (**f, k**); 100 μm (**g–j**); 5 μm (**l–o**).

#### Notes.

Phylogenetically, *Ophiocordycepsmuscidarum* is closely related to *O.globiceps*, with support (100% MLBP, 1.00 PP) (Fig. 1). Base pair differences of LSU, ITS, and *tef-1α* sequences of *O.globiceps* are 4.14% (34/820), 7.43% (35/471), and 2.17% (20/920), respectively. *O.muscidarum* is similar to *O.globiceps* in having a fly (Muscidae, Diptera) as the host, whereas *O.muscidarum* differs from *O.globiceps* in having larger perithecia and longer secondary ascospores (Xiao et al. 2019; Table 3). Additionally, several *Ophiocordyceps* species exhibit a specific affinity for parasitizing dipteran flies. Notable examples include *Ophiocordycepsdipterigena*, *O.globiceps*, *O.hemisphaerica*, and *O.lacrimoidis*, all of which can be distinguished from *O.muscidarum* through phylogenetic analyses (Berkeley and Broome 1873; Freire 2015; Hyde et al. 2016; Xiao et al. 2019). Another species, *Ophiocordycepsdiscoideicapitata*, has also been reported to infect flies but lacks molecular data (Kobayasi and Shimizu 1982). *Ophiocordycepsdiscoideicapitata* differs from *O.muscidarum* in having smaller perithecia and shorter, cylindrical, truncated secondary ascospores (Kobayasi and Shimizu 1982, Table 3). Based on morphology and phylogeny, *Ophiocordycepsmuscidarum* is introduced as a new species in *Ophiocordyceps*.

**Table 3.** Synopsis of a comparison of *O.muscidarum* sp. nov. and its closely related *Ophiocordyceps* species.

Species	Distribution	Stromata (mm)	Perthecia (μm)	Asci (μm)	Ascospores (μm)	Secondary ascospores (μm)	Reference
*O.dipterigena* (First record)	Sri Lanka	5–10 × 1, pale	—	—	—	10 × 1.5	Berkeley and Broome 1873, Freire 2015
*O.dipterigena*	Japan	5–8 × 1–2	700–900 × 240–400	480–600	Filiform, multiseptate	6–12 × 1–1.5, cylindric or fusoid fragments	Kobayasi 1941
*O.dipterigena*	Thailand	4–10	800–1000 × 200–300	450–600 × 4–6	Filiform, breaking up into 64 part-spore	6–12 × 1–1.5, cylindrical to fusiform	Luangsa-ard et al. 2008
*O.dipterigena*	Brazil	9–13 × 0.5–1	850–920 × 230–300	550–700 × 5, filiform	650–700 × 2, 64 part-spores	8–10 × 1–2, cylindrical, fusoids	
*O.discoideicapitata*	Japan	2.5–3.5 × 0.7–1.2	620–700 × 200–250	5–6 diam.	—	6–9 × 1, cylindrical, truncated	Kobayasi and Shimizu 1982
*O.forquignonii*	—	—	—	—	—	Oval, 8	Saccardo 1891
*O.globiceps*	Thailand	4–8 × 0.5–1	538–663 × 182–247	373–454 × 5.7–8	240–303 × 1.8–2.3, filiform	4–5.4 × 1.2–1.9, cylindrical to fusoid	Xiao et al. 2019
*O.hemisphaerica*	Brazil	12–20 × 0.8–1	780–860 × 220–290	500–640 × 5–6	Filiform, more than 52 septa	7–10 × 1–1.5, cylindrical to unusually fusoid	Hyde et al. 2016
*O.lacrimoidis*	Brazil	4–5 × 1	650–700 × 200–250	350–450 × 5	Filiform, more than 56 septa	8–14 × 2, cylindrical	Hyde et al. 2016
***O.muscidarum***	**China**	**5–7 × 1–4**	**570–760 × 190–310**	**280–430 × 5.4–7.5**	**as long as asci, filiform**	**7–10.5 × 1.6–2.5**	**This study**

The symbol “—” means that the data is unavailable.

### ﻿ Ophiocordyceps neocommunis

Taxon classificationFungiHypocrealesOphiocordycipitaceae

Y. Yang, K.D. Hyde & Y. P. Xiao sp. nov.

A99F6CE2-7104-58AB-855C-7A7313D9BE36

Index Fungorum: IF902880

Facesoffungi Number: FoF16767

Fig. 4

#### Etymology.

The epithet “*neocommunis*” refers to the new species’ similarity to its close relative, *O. communis*.

#### Holotype.

China • Guizhou Province, Qiandongnan Miao and Dong Autonomous Prefecture, Rongjiang County, at 382 masl, 25.934°N, 108.479°E, parasitic on termites in soil, 10 June 2023, Yu Yang (HKAS 132236).

#### Description

**(HKAS 132236). *Parasitic*** on termite (Blattodea: superfamily Blattoidea), buried in the soil, the synnemata erect in the air. ***Sexual morph*** not observed in natural substrates and in culture on PDA. ***Asexual morph*: *Hirsutella***-like, the host covered with white mycelium. ***Synnemata*** 3–6 cm long, white to yellow bottom to top. ***Conidiophores*** absent. ***Phialides*** single, borne laterally on synnemata, smooth, hyaline 6.5–12.5 × 3–4.5 µm (*x*–= 9.5 × 3.8 µm, n = 50), basal part strongly swollen, globose, subglobose, or ellipsoid 4.5–8.5 × 3.5–4.8 µm (*x*–= 6.5 × 4.2 µm, n = 50), usually tapering abruptly to a slender neck 0.5–1.2 µm diam. ***Conidia*** 3.0–5.5 × 2.2–4.2 µm (*x*– = 4.2 × 3.2 µm, n = 50), 1-cell, hyaline, oval to teardrop-shaped.

#### Cultural characteristics.

Colonies on PDA grow slowly, reaching 2 cm in diameter after 25 days at 25 °C, ivory yellow, flat, and closely appressed to the agar surface. ***Synnemata*** are produced after 40 days, with the reverse side showing a warm orange. No phialides or conidia found.

**Figure 4.**
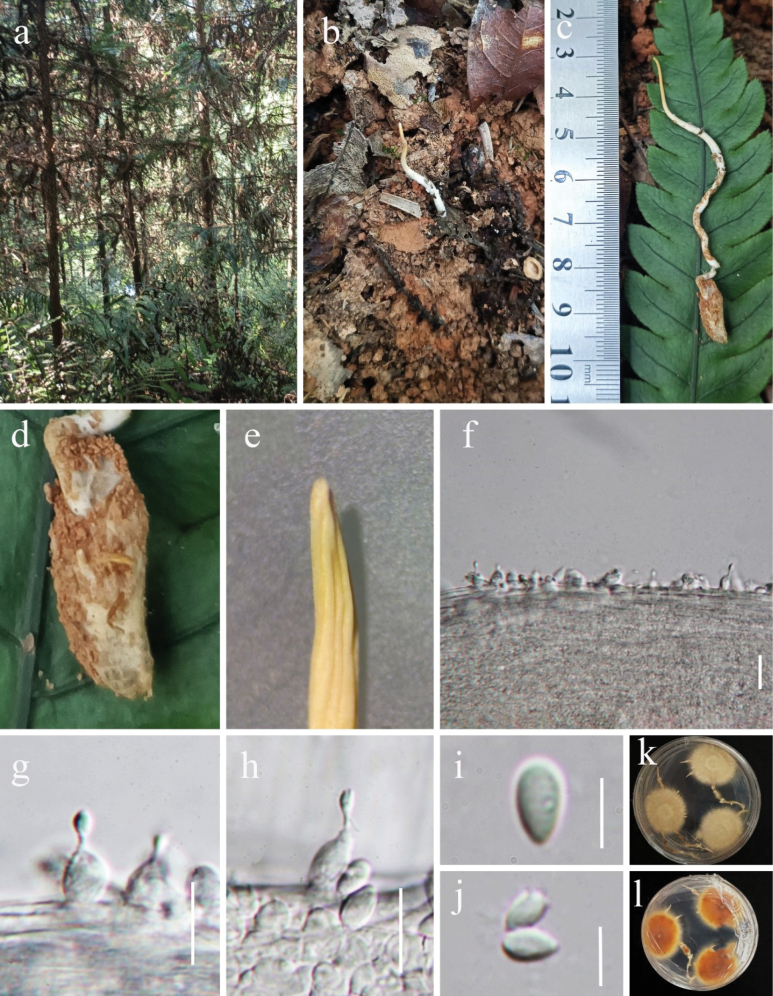
*Ophiocordycepsneocommunis* (HKAS 132236, holotype) **a, b** habitat **c** overview of the fungus **d** host (an unidentified termite species) **e** synnemata **f–h** phialides **i, j** conidia **k–l** culture on PDA. Scale bars: 10 μm (**f–h**); 5 μm (**i–j**).

#### Additional material.

CHINA, Guizhou Province, Qiandongnan Miao and Dong Autonomous Prefecture, Rongjiang County, at 382 masl, 25.934°N, 108.479°E, parasitic on termites in soil, 10 June 2023, Yu Yang, RJ2363J (GZCC 24-0158; ex-type living culture).

#### Notes.

*Ophiocordycepsneocommunis* clustered with *O.communis* and *Hirsutellaminnesotensis* in the phylogenetic tree, supported by 100% MLBP and 1.00 PP (Fig. 1). Notably, the differences between *O.communis* and *H.minnesotensis* are minimal, as indicated by the short branch length in the phylogenetic tree. However, further evidence is needed to determine whether they represent the same species. *Ophiocordycepscommunis* shares its host, termites (Blattodea, superfamily Blattoidea), with *O.neocommunis* but differs in the morphology of its phialides. Specifically, *O.communis* produces longer and narrower phialides, as well as longer conidia (Sung et al. 2007b; Table 4). *Hirsutellaminnesotensis* is distinct from *O.neocommunis*, as it is isolated from second-stage juveniles of the soybean cyst nematode and has longer phialides (9–15 µm vs. 6.5–12.5 µm) (Chen et al. 2000). Comparing the ITS, *tef-1α*, *rpb1*, and *rpb2* sequences of *Ophiocordycepsneocommunis* and *O.communis* revealed 98.83% (6 bp differences), 97.57% (22 bp differences), 98.59% (10 bp differences), and 98.76% (13 bp differences) sequence similarities, respectively. Comparing the ITS sequences of *Ophiocordycepsneocommunis* and *Hirsutellaminnesotensis* revealed 98.83% (6 bp differences). Thus, we would like to introduce *Ophiocordycepsneocommunis* as a new species based on phylogenetic and morphological analyses.

**Table 4.** Synopsis of a comparison of *O.neocommunis* sp. nov. and its closely related *Ophiocordyceps* species.

Species	Distribution	Asexual morph	Phialides (µm)	Conidia (µm)	Reference
*O.asiatica*	Thailand	*Hirsutella*-like	15–20 × 2–3	Fusiform, 7–9 × 2–3	Tasanathai et al. 2019
*O.brunneirubra*	Thailand	*Hirsutella*-like	32–50 × 2–3	Fusiform, 12–17 × 2–4	Tasanathai et al. 2019
*O.communis*	Thailand	*Hymenostilbe*/*Hirsutella*-like	10–14 × 2.7–3.3	Almond-shaped, 7–9 × 2.5–3	Sung et al. 2007a
*O.fusiformis*	Thailand	*Hymenostilbe*-like	9–24 × 2–4	Fusiform, 6–18 × 2–4	Tasanathai et al. 2022
*O.globosa*	Thailand	*Hirsutella*-like	9–15 × 3–5	Globose, 2–4	Tasanathai et al. 2022
*O.isopterae*	Thailand	*Hirsutella*-like	14–28 × 2–4	Fusiform, 6–11 × 1.5–3	Tasanathai et al. 2022
*O.khokpasiensis*	Thailand	*Hirsutella*-like	15–28 × 3–5	Globose to oval, 4–6 × 2.5–4	Khonsanit et al. 2019
*O.longistipes*	China	*Hirsutella*-like	29–60 × 4–4.5	Citriform or oval, 7–10 × 4.5–7	Fan et al. 2024
*O.mosingtoensis*	Thailand	*Hirsutella*-like	10–17 × 2–3	Oval, 3–5 × 2–3	Tasanathai et al. 2019
***O.neocommunis***	**China**	***Hirsutella*-like**	**6.5–12.5 × 3–4.5**	**Oval to teardrop-shaped 3.0–5.5 × 2.2–4.2**	**This study**
*O.ovatospora*	China	*Hirsutella*-like	15–35 × 3–6	Oval, 3–5 × 3–4	Tang et al. 2022
*O.pseudocommunis*	Thailand	*Hymenostilbe*-like	17–22 × 2–8	Fusiform, (6–)6.5–7.5(–8) × 2–3	Tasanathai et al. 2019
*O.pseudorhizoidea*	Thailand	*Hirsutella*-like	9–21 × 2–4	Fusiform, 5–10 × 1–2	Tasanathai et al. 2019
*O.puluongensis*	Vietnam	*Hirsutella*-like	7.9–21.2 × 1.7–5.0	Fusiform or citriform, 2.8–6.1 × 1.9–3.4	Xu et al. 2022
*O.radiciformis*	Thailand	*Hirsutella*-like	6–15 × 2–5	Fusiform, 5–7 × 2–3	Tasanathai et al. 2022
*O.termiticola*	Thailand	*Hirsutella*-like	7–11 × 2.5–4	Globose, 2.5–3.5	Tasanathai et al. 2019

### ﻿ Hirsutella vermicola

Taxon classificationFungiHypocrealesOphiocordycipitaceae

M.C. Xiang & Xing Z. Liu, in Xiang, Yang, Xiao, Liu & Chen, Fungal Diversity 22: 258 (2006)

15379E91-48FB-508B-BF4A-7DCE2F09A7F0

Index Fungorum: IF500924

Fig. 5

#### Description

**(HKAS 132167). *Parasitic*** pupa of Lepidoptera, buried in soil. ***Sexual morph*** not observed in natural substrates. ***Asexual morph*: *Synnemata*** 2–6 cm long, 1–3 mm wide, cylindrical with tapering tip, stipitate, gradually become white or green yellowish to the upper portion, scattered on the insect head, without fertile head. ***Phialide*** solitary along synnema, 15.2–25.5 × 1.3–4.8 µm (*x*–= 20.3 × 3.1 µm, n = 60), solitary, upright, typically cylindrical, with a wider base that gradually narrows towards the top 7.2–15.3 × 2.4–4.8 µm (*x*–= 11.2 × 3.6 µm, n = 60), tapering into a long neck 7.5–11.5 µm long, has a rough surface, featuring verrucose projections. ***Conidia*** 4.6–6.8 × 2.2–3.2 µm (*x*– = 5.7 × 2.7 µm, n = 60), fusiform or oval, 1-celled, hyaline.

#### Material examined.

China • Guizhou Province, Zunyi City, Xishui National Nature Reserve, at 1069 masl, 28.494°N, 106.388°E, parasitic on pupa of Lepidoptera, buried in soil, 10 April 2023, Yu Yang, XS2310, XS2311 (HKAS 132167, HKAS 132168).

**Figure 5.**
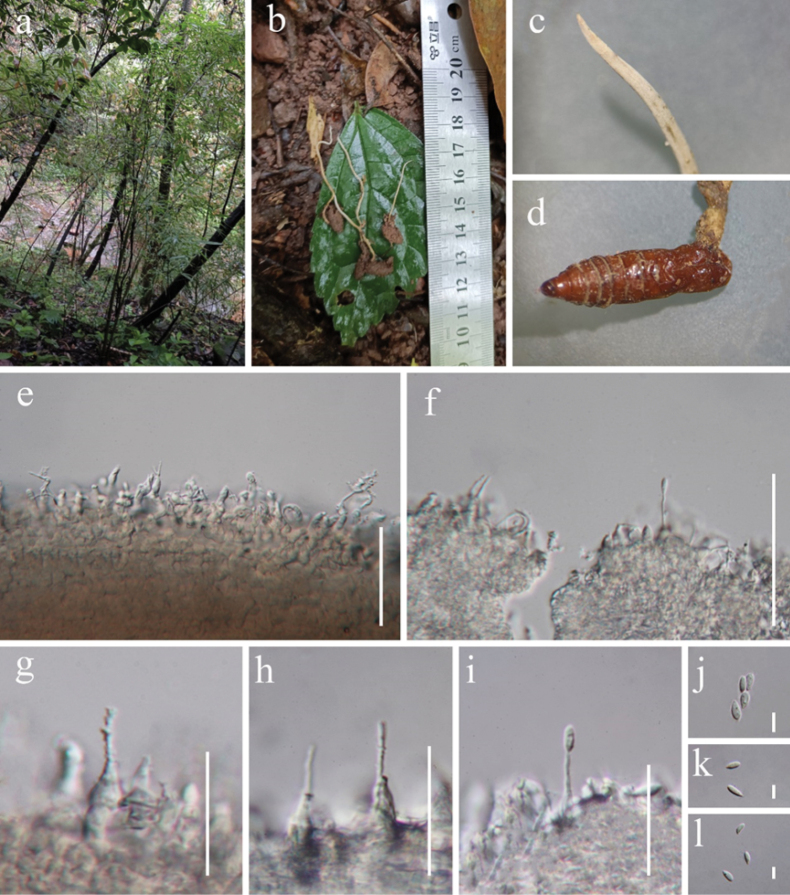
*Hirsutellavermicola* (New host, HKAS 132167) **a** habitat **b** synnemata on host **c** synnema **d** host (pupa of Lepidoptera) **e–h** phialides **i** phialides with conidia **j–l** conidia. Scale bars: 50 µm (**e–f**); 20 µm (**g–i**); 5 µm (**j–l**).

#### Notes.

*Hirsutellavermicola*, introduced by Xiang et al. (2006), was found in bacteria-feeding nematodes in the United States. Phylogenetic analysis showed that our new isolates, HKAS 132167 and HKAS 132168, are nested with *Hirsutellavermicola* (CGMCC 3.7877, AS3.7878, and AS3.7879) (Fig. 1). Morphologically, our new isolate is almost identical to *Hirsutellavermicola* except for the conidia (Xiang et al. 2006). Our new isolate has smaller conidia than the holotype of *H.vermicola* (4.6–6.8 µm vs. 6–8 µm) (Xiang et al. 2006). The molecular data of our new isolate (HKAS 132168) are not significantly different from those of *Hirsutellavermicola* (CGMCC 3.7877, AS3.7878, and AS3.7879). Thus, we identified our new isolate as *H.vermicola*. This is the first report of *H.vermicola* parasitic on the pupa of Lepidoptera, which broadens the range of hosts. Furthermore, this is the first report of *H.vermicola* in China.

## ﻿Discussion

The taxonomy of entomopathogenic fungi has undergone significant revisions in the molecular era, moving toward a monophyletic classification (Tasanathai et al. 2016; Dong et al. 2022). Initially, ITS sequences were inadequate for differentiating closely related species due to their limited variability (Chen et al. 2002; Liu et al. 2002; Stensrud et al. 2005). To enhance phylogenetic resolution, additional genes were incorporated into analyses, including the ribosomal small subunit (SSU), large subunit (LSU), and protein-coding genes such as the elongation factor 1-alpha (*tef-1α*), RNA polymerase II largest subunit (*rpb1*), second largest subunit (*rpb2*), and β-tubulin (TUB). These combined analyses provided more comprehensive phylogenetic insights (Sung et al. 2007b; Khonsanit et al. 2020; Fan et al. 2024). The adoption of Genealogical Concordance Phylogenetic Species Recognition (GCPSR) has since improved species identification and classification within the *Ophiocordyceps*, leading to more accurate taxonomic delineations (Sung et al. 2007b; Khonsanit et al. 2020; Mongkolsamrit et al. 2021). As genomic sequencing of *Ophiocordyceps* species has expanded, our understanding of their phylogenetic relationships and taxonomic framework has deepened, deepening our understanding of their phylogenetic relationships and taxonomic framework (Wang et al. 2016; Shu et al. 2020; de Menezes et al. 2023).

*Ophiocordyceps* exhibit remarkable parasitic adaptability, infecting a wide range of hosts. Our research has identified new species that parasitize insects from the orders Blattodea, Coleoptera, Diptera, and Lepidoptera. Termites, particularly those from the superfamily Blattoidea, are also known hosts for some *Ophiocordyceps* species (Tasanathai et al. 2019; Tasanathai et al. 2022; Fan et al. 2024). Despite the global presence of over 300 *Ophiocordyceps* species, fewer than 19 are reported to parasitize termites, with occurrences documented in diverse regions including China, Indonesia, Japan, Kenya, Mexico, Tanzania, and Thailand (Stifler 1941; Blackwell and Gilbertson 1981; Ochiel et al. 1997; Tasanathai et al. 2019; Tasanathai et al. 2022; Fan et al. 2024). Termite-infecting *Ophiocordyceps* typically reside 5 to 15 cm underground, mirroring the subterranean habits of their termite hosts (Martelossi et al. 2023). However, identifying these fungi poses challenges due to the potential loss of fragile stromata during excavation (Tasanathai et al. 2022). In China, four termite-associated species have been identified: *Ophiocordycepslongistipes*, *O.globiperitheciata*, *O.ovatospora*, and *O.puluongensis* (Tang et al. 2022; Xu et al. 2022; Fan et al. 2024). Comparatively, *Ophiocordycepsneocommunis* features white to yellow synnemata, with strongly swollen basal part phialides, and single, oval to teardrop-shaped conidia.

The taxonomic distinction between *Ophiocordyceps* and *Hirsutella* remains unclear, as evidenced by their recurrent clustering in phylogenetic trees (Evans and Samson 1982; Quandt et al. 2014; Spatafora et al. 2015). This study, which introduces three new *Ophiocordyceps* species and a new host record for *H.vermicola*, underscores the need for further phylogenetic clarification, as our phylogenetic tree also shows that both genera are clustered together. Extensive research has demonstrated the widespread parasitism of both genera across a variety of insect hosts (Spatafora et al. 2015; Araújo et al. 2018; Xiao et al. 2019; Mongkolsamrit et al. 2021; Tasanathai et al. 2022; Yang et al. 2024). The three new species and the new host record identified in our study further reinforce this pattern, with hosts spanning multiple insect orders, families, and genera, including flies, lepidopteran pupae, termites, and wireworms (Tasanathai et al. 2019; Zha et al. 2021). Importantly, the adaptation of *Ophiocordyceps* and *Hirsutella* species to specific hosts underscores the influence of host diversity on the genetic diversity and geographic distribution of both genera.

*Ophiocordyceps* species in the unilateralis complex exhibit remarkable specificity, targeting exclusively the ant genus *Camponotus*, which significantly alters the ant’s behavior to facilitate its reproduction (de Bekker et al. 2014; Araújo et al. 2018). Similarly, *Ophiocordycepssinensis* has a specialized association with ghost moth larvae (*Thitarodes* spp.), parasitizing the larvae to produce the medicinally valuable fungus known as “yarsagumba” (Baral 2017). Furthermore, Somavilla et al. (2019) investigated how *Ophiocordycepshumbertii* influences the behavior of social wasps in Neotropical forests, highlighting the fungus’s adaptability and its impact on its host. These instances underscore how *Ophiocordyceps* species adapt to specific hosts, influencing their genetic diversity and distribution across ecosystems.

Advancements in phylogenetic research and taxonomy, particularly through DNA sequencing, are crucial for accurately identifying and classifying *Ophiocordyceps* and *Hirsutella* species (Tasanathai et al. 2016; Xiao et al. 2019; Dong et al. 2022; Yang et al. 2024). Moreover, the potential of *Ophiocordyceps* in pest management is increasingly recognized. For example, *Ophiocordycepsunilateralis* regulates ant populations by infecting and controlling their behavior, which helps protect crops from ant damage (Tasanathai et al. 2019; Moon et al. 2023; Fan et al. 2024), and *O.nutans* is being developed as a natural medicine and biopesticide to protect crops and forest trees (Paul et al. 2020). Utilizing *Ophiocordyceps* in biological control highlights its significant potential for promoting sustainable agriculture and protecting ecological environments. In this study, three new species, *Ophiocordycepsmuscidarum*, *O.neocommunis*, and *O.liaoningensis*, and one new host record for *Hirsutellavermicola*, associated with insects from China, were introduced based on phylogenetic inferences of a combined LSU, ITS, SSU, *tef-1α*, *rpb1*, and *rpb2* DNA sequence dataset and morphological evidence.

## Supplementary Material

XML Treatment for Ophiocordyceps liaoningensis

XML Treatment for Ophiocordyceps muscidarum

XML Treatment for Ophiocordyceps neocommunis

XML Treatment for Hirsutella vermicola

## ﻿Additional information

### Author ORCIDs

Yu Yang https://orcid.org/0000-0001-8268-487X

Ruvishika S. Jayawardena https://orcid.org/0000-0001-7702-4885

Kevin D. Hyde https://orcid.org/0000-0002-2191-0762

Yong-Zhong Lu https://orcid.org/0000-0002-1033-5782

Fatimah Al-Otibi https://orcid.org/0000-0003-3629-5755

### Data availability

All of the data that support the findings of this study are available in the main text.
